# Angioneurotic oedema with tadalafil: A rare case report

**DOI:** 10.4103/0019-5545.31562

**Published:** 2006

**Authors:** Rajnish Raj, Balwant Sinch Sidhu

**Affiliations:** * Senior Resident Department of Psychiatry, Government Medical College and Rajindra Hospital, Patiala; ** Additional Professor Department of Psychiatry, Government Medical College and Rajindra Hospital, Patiala

**Keywords:** Tadalafil, ED (erectile dysfunction), BSFI (Brief Sexual Function Inventory) scale, GAF (Global Assessment of Functioning) scale

## Abstract

A case is reported where the patient developed angioneurotic oedema of the lip after the use of tadalafil.^1^ On withdrawal of tadalafil, complete remission was obtained through required symptomatic treatment. Clinicians should be aware of the possibility of development of angioneurotic oedema in patients undergoing treatment with tadalafil.

## INTRODUCTION

Tadalafil is a reversible inhibitor of cyclic guanosine monophosphate (cGMP) specific phosphodiestrase PDE5 enzyme used in the treatment of erectile dysfunction. There is a report in the literature regarding emergence of symptoms of pruritis, rash, sweating and facial oedema.^2^ This article reports a case of a patient who presented with symptoms of angio-neurotic oedema of the lip after the use of tadalafil.

## THE CASE

A 30-year-old, postgraduate, married man reported to the Department of Psychiatry, Government Medical College and Rajindra Hospital, Patiala. He complained of decreased libido and recurrent inability to attain or maintain erection to complete the sexual act and denied having any problem of arousal, premature ejaculation and orgasmic dysfunction for the past 6 months. In the sexual history, including the number of erections, time intervals and quality of erection, the performance and level of satisfaction was inadequate; he scored 34 and 69 as assessed on the Brief Sexual Function Inventory (BSFI) and Global Assessment of Functioning (GAF) scales respectively, which showed that there was mild functional impairment due to erectile dysfunction.

The patient had a normal masturbatory activity during solitary hours with adequate erections. The erectile dysfunction was not better accounted for any other axis–I disorder (e.g. major depressive disorder, obsessive–compulsive disorder, etc.). It was also not due to the direct physiological effects of a substance (e.g. drug of abuse or medication) or a general medical condition. There was no history of hypertension, diabetes, surgical intervention, spinal injury or any other medical illness. There was no personal and family history of any allergy or autoimmune disorder and had a well-adjusted, premorbid personality.

Physical, neurological and mental status examination was normal.

### Laboratory examinations

Hb (14.5 g%), TLC/DLC (9700 /cmm; 67/28/3/1), ESR (8 mm/hour Wintrobe method), Na (138 mEq/L; K 3.9; chloride 102), blood urea (30 mg/dl), serum creatinine (0.7 mg/dl), serum bilirubin (0.7 mg/dl), alkaline phosphate (9 KAU), urine (total, albumin, globulin 6.9, 3.3, 3 g/dl), stool examination (NAD). Endocrinal (testosterone 10 nmol/L), dihydrotestosterone (1.6 ng/ml), FSH/LH (15 mU/ml), cortisol (450 nmol/l AM, 280 nmol/l PM), post-dexamethosone (<150 nmol/L), prolactin (77 mU/L), T4 (66 ng/ml), T3 (0.9 ng/ml), TSH (5 mU/ml) were within normal limits.

Specific tests such as nocturnal penile tumescence and colour doppler test for vascular insufficiency were normal.

The diagnosis of male erectile dysfunction, acquired, psychogenic type was made as assessed on DSM-IV TR. The patient was prescribed tadalafil 10 mg once daily for 7 days along with individual psychotherapy. After the start of therapy on day 1 within 30 minutes, the patient reported headache, nasal stuffiness. On day 3 after an hour of taking tadalafil, the patient complained of heaviness and slight swelling on the right side of the upper lip which gradually swelled within 15 minutes to involve the whole of the upper lip with eversion; the lower lip was spared, followed by development of red pruritic rash over the neck and front of the chest. There was no history of pharyngeal, laryngeal or bronchial oedema. The patient denied taking any food preservatives other than the routine diet. There was no change of toothpaste, after-shave or any dental problem. He was not taking any nasal decongestant or any other drug which could cause allergy. There was no history of worm infestation, insect bite, exposure to pollen, animal dander, cold and heat intolerance, substance abuse or any autoimmune disorder (hereditary angioneurotic oedema type I and II).

The patient reported to the Emergency Department of Psychiatry, where he was given inj. avil 50 mg and inj. hydrocortisone 200 mg intravenously. The provisional diagnosis of drug-induced angioneurotic oedema was made and he was referred to the Department of Dermatology. The patient was investigated and the laboratory tests were as follows: Hb (14 g%), TLC/DLC (8700/cmm; 60/28/1/1), ESR (4/mm/hour Wintrobe method), Na (136 mEq/L; K 3.9; chloride 102), blood urea (29 mg/dl), serum creatinine (0.6 mg/dl), serum bilirubin (0.65 mg/dl), alkaline phosphate (9 KAU), urine (total, albumin, globulin 6.5, 3.0, 2 g/dl) and stool examination (NAD) which were within normal limits. After exploring the drug history, it was diagnosed a case of drug-induced angioneurotic oedema of the lip. Tadalafil was stopped and he was prescribed Tab. levocetrizine 10 mg once daily and Tab. ebastine 5 mg at bedtime for 7 days. On day 7, ebastine was stopped and levocetrizine was gradually tapered off. The symptoms of angioneurotic oedema of the lip gradually subsided.

After 2 months, he was again prescribed tadalafil 10 mg for erectile dysfunction and presented with swelling of the lower lip, reddish pruritic rash over the upper torso and flexor areas of the upper as well as lower limbs within 30 minutes of taking the drug. The patient took treatment from the Department of Dermatology, and tadalafil was stopped and the patient recovered.

## DISCUSSION

Angio-oedema^3^ is a variant of urticaria. Sites involved are lips, eyelids, genitalia, tongue and pharynx.^4^ Drug-induced urticaria is seen in 0.16% of medical inpatients and it accounts for 9% of chronic urticaria or angio-oedema seen in dermatology outpatients.^5^ Occurring within 24–36 hours of drug ingestion, urticaria is seen in association with anaphylaxis, angio-oedema and serum sickness. On re-challenge, lesions may develop within minutes. Angio-oedema involving oedema of the dermis, subsuctaneous and submucosal areas, is more rarely seen than urticaria as an acute drug reaction, and occurs in less than 1% of the patients receiving the particular drug.^6^

Although tadalafil (a reversible CGMP PDE5 enzyme inhibitor) has been reported to be associated with various side-effects such as headache, dyspepsia, back pain, myalgia, nasal congestion, flushing, pain in limbs, etc., yet it is considered a safe drug with good tolerance and favourable pharmacokinetics. It is possibile that in this case it was drug-induced angioneurotic oedema of the lip. The clinical symptoms appeared within a day after starting tadalafil. However, it remitted when the drug was withdrawn and symptomatic treatment was initiated. The gradual remission in case of tadalafil was due to prolonged plasma half-life of the drug.^1^ The case report highlights the development of allergic rash^2^ as a rare presentation and recommends further research on the psychopharmacological basis of newer drugs and its variability in ethnic groups.
